# Risk factors underlining reproductive performance in smallholder beef cattle herds of South Africa

**DOI:** 10.1007/s11250-024-04181-x

**Published:** 2024-10-02

**Authors:** Marble Nkadimeng, Este Van Marle-Köster, Nkhanedzeni B. Nengovhela, Fhulufhelo V. Ramukhithi, Masindi L. Mphaphathi, Johannes M. Rust, Mahlako L. Makgahlela

**Affiliations:** 1 Department of Animal and Wildlife Sciences, University of Pretoria, Hatfield, Pretoria, 0002 South Africa; 2 Agricultural Research Council, Germplasm Conservation and Reproductive Biotechnologies, Private Bag X2, Irene, Tshwane, 0062 South Africa; 3 Department of Agriculture, Land Reform and Rural Development, Delpen Building, Corner Annie Botha, and Union Street, Riviera, Pretoria, 0001 South Africa; 4 Department of Agriculture and Animal Health, University of South Africa, Florida, 1710 South Africa; 5 Döhne Agricultural Development Institute, Stutterheim, 4930 South Africa; 6 Department of Animal, Wildlife and Grassland Sciences, University of the Free State, Bloemfontein, 9301 South Africa

**Keywords:** Pregnancy rate, Reproductive management, Reproduction efficiency, Herd performance

## Abstract

Improving the historically low reproductive performance of beef cattle in smallholder systems is essential for its productivity and profitability. Therefore, identifying and addressing risk factors associated with low performance in this system present an opportunity for improvement. The study aimed to evaluate the effect of animal and management risk factors on pregnancy rate (PR), fetal and calf loss (FC), calving interval (CI) and days open (DO) in smallholder beef cattle farms. A multi-stage selection approach was conducted in five provinces of South Africa. Cow records (3694) collected from 40 smallholder herds over two years (2018–2019) were analysed. Data on animal and herd management factors including body condition score (BCS), cow age class, breed type, lactation status, culling old/non-productive cows, record-keeping, and breeding and calving seasons were recorded. The GLIMMIX procedure was computed to determine risk factors associated with performance indicators (PR, FC, CI and DO). Risk factors highly associated with performance were breed type, BCS, cow-age class and breeding/calving season (*P* ≤ 0.05). Indicators FC, DO and CI increased with decreased BCS, autumn calving seasons, and in first calvers and aged cows. Whereas, PR increased with increasing odds of BCS and breeding seasons between December-March, November-February and January-March. Optimizing reproductive performance in smallholder beef cattle herds may involve managing cow body condition, breeding with adapted genotypes and synchronizing breeding with favourable seasons. Therefore, record keeping and regular monitoring of herd nutrition, climate and breed performance may be significant in improving reproductive performance in smallholder herds.

**Supplementary Information:**

The online version contains supplementary material available at 10.1007/s11250-024-04181-x.

## Introduction

Over decades, multiple reports in South Africa (SA) have provided extensive evidence of low reproductive performance in smallholder beef cattle herds (Mokantla et al. 2004; Nowers et al. 2013; Mugwabana et al. 2018). These studies have recommended the need for acquiring comprehensive knowledge on potential underlying factors that influence reproductive performance of beef cattle in low-input farming systems (Mokantla et al. 2004; Tada et al. 2013; Van der Westhuizen et al. 2020). The importance of factors influencing reproductive performance has been studied in countries such as Bangladesh, Brazil and Indonesia (Khan et al. 2015; Kaurivi et al. 2021; Reis et al. 2023). These studies successfully identified constraining factors which led to management strategies that positively impact fertility traits. Evidence in the aforementioned countries showed a 14% increase in pregnancy rates, 64% increase in calving rate and improved calving intervals from days 507 to 486 (Khan et al. 2015; Ratnawati et al. 2016; Reis et al. 2023). Reproduction is closely associated with farm profit and herd growth (Tadesse and Tegegne 2018). The low reproductive performance in smallholder beef cattle farms is a critical issue which impacts the estimated 2.5 billion rural communities in Africa that depend on its success for rural economic growth, food security and overall development (FAO 2019).

In recent studies, Mugwabana et al. (2018) and Nengovhela et al. (2021) emphasized the importance of factors influencing herd reproduction by modeling the probability of calving rates for communal farms in South Africa. While their research was informative, their conclusions were limited to one-dimensional approach, focusing solely on the calving rate trait. Whereas, considering multiple risk factors on multiple traits may provide an understanding of the interconnections and complexities influencing fertility traits in smallholder beef cattle herds (Giordano et al. 2022). A holistic management approach can be particularly beneficial for resource constraint farmers in finding a unified management approach for diverse reproductive performance to improve poor herd reproduction (Terry et al. 2020). For example, studying body condition scoring (BCS) of breeding cows on multiple fertility traits concurrently may positively affect the fertility performance of animals resulting in shorter calving intervals, increased pregnancy rates and reduced days open (Fernandez-Novo et al. 2021; Nazhat et al. 2021).

Important fertility traits including PR, DO and CI are reported to be influenced by variety of animal and management risk factors such as BCS, cow age, lactation status, breeding season, breed type and record keeping (Burns et al. 2010; Temesgen et al. 2022; Copley et al. 2022). Considering these factors, smallholder farmers may expand knowledge in improving reproductive performance and set targeted strategies to address challenges in their production systems (Montiel-Olguín et al. 2019). These strategies may involve breeding with adaptive breeds to reduce reproductive failures from environmental stressors, implementing age-specific nutritional needs for proper feeding programs and managing the postpartum anestrous period associated with extended lactation (D'Occhio et al. 2019; Cooke et al. 2020; Reis et al. 2023). The present paper forms part of a three-part series of research papers aimed at addressing the question of "*what breeding systems need to be developed and implemented to cost-effectively improve reproduction performance in the smallholder beef cattle sector*." The first paper provided an understanding of smallholder beef cattle farming and its associated challenges (Nkadimeng et al. 2022a), while the second paper defined a set of indicators and established benchmarks for reproductive performance (Nkadimeng et al. 2022b). The current paper is the third in the series. The study aimed to employ a multistage analysis approach to investigate animal and management risk factors associated with PR, FC, CI and DO in smallholder beef cattle farms of SA. Understanding these factors will contribute knowledge for improvement interventions of reproductive performance in SA beef cattle smallholder herds.

## Material and methods

### Ethical approval and sampling procedure

**Fig. 1:**
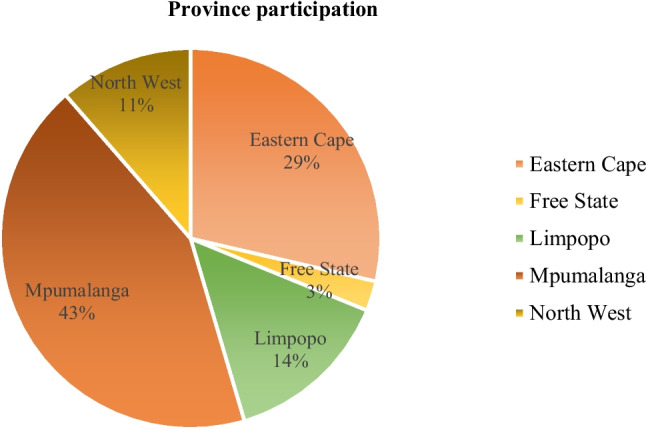
Distribution of study herds across provinces. Percentage of participating herds across the five provinces (Eastern Cape, Free State, Limpopo, Mpumalanga and North West)

Ethical approval for the current research was obtained from the Ethics Committee (AEC) of the University of Pretoria (NAS339/2020). A comprehensive description of the methods and study provinces used in this research has been provided by Nkadimeng et al. (2022b). Briefly, the study employed a multi-stage sampling strategy to collect performance data from five provinces participating in the High Value Beef Partnerships (HVBP) project (LS-2016–276) (Eastern Cape, Free State, Limpopo, Mpumalanga, and North West). Selection of the provinces was based on their contractual involvement in the HVBP project. Herds were purposefully selected based on the availability of handling facilities enabling collection of key parameters such as pregnancy diagnosis. The participation of herds per province are illustrated in Fig. 1. Breeding cows were selected with the criterion that they had calved before.

### Herds and animals

The study collected a total of 3694 cow records from 40 herds during the years 2018 and 2019. Each herd underwent biannual visits: in Autumn season (March–May) for pregnancy diagnosis and Spring season (September–November) to track confirmed pregnancies, record pregnancy losses, and detect new pregnancies. Briefly, in 2018, data were collected from 16 herds. Of the 16 herds recorded, only five were repeated in 2019 with an addition of 19 new herds. This resulted in a total of 24 repeated herds recorded in 2019. The remaining 11 herds that were not repeated in 2019 were excluded from the project due to their non-compliance with project requirements related to either market specifications or herd health challenges (Nkadimeng et al. 2022b).

During farm visits, cows underwent physical examinations to assess their lactation status, body condition score and measuring of the hip height. The BCS was assessed using a standardized body condition scoring systems of 1–5 and lactation status were recorded as ‘wet’ or ‘dry’ through expressing milk from the teats. To measure hip height, a retractable measuring tape was lowered vertically from a set distance above the squeeze chute and the measurement was taken from the fixed point to the highest part of the animal's back between its hips. Additionally, cow age class (first calvers, second calvers, matured and old cows) detailed in McGowan et al. (2014), as well as the breed type for each cow were recorded (Table 5.2; Table S1).

### Measurements collected

Pregnancy rate (PR), fetal and calf loss (FC), days open (DO) and calving interval (CI) were fertility indicators measured. Pregnancy rate indicator was calculated as the proportion of cows confirmed pregnant to the total number of cows tested for pregnancy within the participating herds. A portable ultrasound scanner [monitor (Ibex pro, EI medical imaging, USA; transducer (5 MHz/12 cm depth)] was used to diagnose pregnancy in participating herds. For each pregnant cow, gestation length or fetal age was recorded in months. Indicator FC for this study was defined as the percentage of both abortion and calf mortality in the herd. The FC was measured on cows that were initially identified as pregnant at the start of pregnancy diagnosis (March–May) however, open and not lactating at the time of the final pregnancy diagnosis (September–November). The number of days between calving and conception were used to define indicator DO. While, the number of days between two consecutive calving were used to define CI. For each participant cow, the age of the last calf and gestation duration of each participating cow were used to estimate DO and CI. The variables DO and CI were divided into four groups [acceptable (121; 365), concern (182: 425), extended (243:456), and overly extended (304:604)] for better understanding of the heterogeneity within smallholder farms (Nkadimeng et al. 2022b). All 3694 records collected from 40 herds were pooled to measure PR. Indicators DO, CI and FC were assessed on 1401 records from 24 repeated herds (Autumn and Spring season collection).

### Risk factors assessed

Animal risk factors recorded for each participating cow included BCS, hip height, lactation status, cow age class and breed type. The various breed types were distinguished based on their physical characteristics and resemblances (as shown in Table S1). Herd management factors (e.g. knowledge of the body condition score (BCS) prior to breeding, culling of old and none productive cows, breeding season, calving season, record-keeping, age of the last calf and bull to cow ratio) were all recorded through questionnaire guided interviews with each farmer (Table 1).

**Table 1:** The description of animal and herd-level risk factors considered in the multivariable model building process

Risk factors	Description
**Animal level**	
Breed type	Type of breed identified in smallholder farms
Cow age class	First calvers: cows nursing their first calf; second calvers: cows weaned their first calf, matured cows: cows age between 5–7 years, aged cows: cows over 8 years
BCS at breeding	Body condition scoring ranged from 1 = lean animal to 5 = obese
BCS at calving	BCS scoring ranged from 1 = lean to 5 = obese
Lactation status	1 = Wet, 2 = Dry during pregnancy diagnosis
Hip height	Short (< 125 cm), Moderate (125 to < 140 cm) and Tall (≥ 140 cm)
**Management**	
Breeding season (Breeding months recorded according to each farmer's practices on their herds)	January–March, March–June, August–October, September–December, November–February and December–March. Continuous
Calving season	Spring, Summer, Autumn, Winter
Reasons for calf losses	1 = Abortions, 2 = Stillbirth
Culling non-productive cows	1 = yes, 2 = no
BCS prior breeding season	1 = yes, 2 = no
Bull to cow ratio	1 = Ideal = (1:30), 2 = Under = (1:15) and 3 = Over = (1:70)
Age of the last calf	Age in months of young calf for each breeding cow
Records keeping	1 = yes, 2 = no

The animal and herd-level risk factors were collected simultaneously during on farm visits. BCS at breeding: Body condition score recorded during breeding season; BCS at calving: Body condition score recorded during calving season. Continuous breeding season is breeding season throughout the year

### Statistical analysis

Data were analysed using Statistical Analysis System (SAS) 9.4. Descriptive statistics for average levels of performance was obtained using frequency tables. A multilevel logistic regression model with random effects was applied using GLIMMIX procedure of SAS 9.4 to assess measures of association between animal and management risk factors with performance indicators (PR, FC, CI and DO). The chi-square test was employed to evaluate the presence of collinearity among the covariates (animal and management risk factors) yielding a Cramer V statistic of > 0.6. In cases where collinearity occurred between variables, only one variable was fitted into the model. The model incorporated provinces as random effects while risk factors were incorporated as fixed effects. Farms were treated as the experimental units. An empty unconditional model without any predictors served as the starting point for the modeling procedure. This model provided a general estimation of the reproductive performance (PR, FC, CI, and DO) for farms at a typical province and information regarding the performance variation between provinces. The intraclass correlation coefficient (ICC) assessed the extent to which province, animal and management factors contribute to the overall variations in reproductive performance (PR, FC, DO, and CI). The ICC was evaluated as follows:

$$ICC=\frac{{\beta }_{0}}{{\beta }_{0} +3.29}$$

where ${\beta }_{0}$ is the covariance parameter estimate and 3.29 was used the level-1 error variance in calculating the ICC as proposed by Ene et al. (2015).

The model-building process continued to include risk factors as fixed effects while controlling for provinces to estimate factors associated with performance measures at a national level. A cumulative ordinal regression procedure was conducted for the CI and DO indicators, while a binary logistic regression procedure was used for PR and FC indicators to determine the risk factors associated with reproductive performance. The binary model was described as follows:

$$In\left(\frac{P\left({Y}_{ij=1}\right)}{\left({Y}_{ij=0}\right)}\right)={a}_{i} +{\beta }_{xij}+{u}_{ij}$$

${Y}_{ij}$ is the binary indicator of the ${i}^{th}$ farm in the ${j}^{th}$ province, with ${Y}_{ij}$ = 1 representing the probability of success (pregnancy/loss) and ${Y}_{ij}$ = 0 indicates failure. The ${a}_{i}$ is the intercept and the regression coefficient of the ${x}_{ij}$ covariates is represented by$\beta$. Furthermore, ${u}_{ij}$ is the random effect representing the effect of the ${j}^{th}$ province.

The cumulative logit procedure simultaneously estimates multiple equations for the comparison of the cumulative odds of high versus low CI and DO categories. For this study, the predictor variable CI and DO had four categories as follows.

$$j=\left\{\begin{array}{c}accepted\\ concern\\ extended \\ overly extended\end{array}\right.$$

where overly extended category represent high outcome category and accepted category represent low outcome category.

Therefore, the logits regression model used for CI and DO was defined as:

$$\left(\frac{P\left(Y\ge j\right)}{1-P\left(<j\right)}\right)={a}_{j+{B}_{x }+{u}_{j, }} \left(j=(\text{1,2}\dots \dots j-1)\right)$$

where $P\left(Y\ge j\right)$ is the odds of the event of the category $j$ of a given predictor variable (CI and DO); ${a}_{j}$ is the intercept parameter and $\beta$ is the vector of regression coefficients corresponding to $x$ covariates. The model specifies that the intercept parameter differs across all $j$ categories however, the $x$ covariates remain constant. The odds of the highest $j$ level category (overly extended) was used to compare with the lower level category (accepted).

## Results

Descriptive statistics of performance indicators are presented in Table 2. Smallholder farms recorded an overall pregnancy rate of 50%. Of this, Bonsmara breed type (20.46%) and mature cows (33.62%) of BCS of 3 (27.73%) reported majority of PR. The majority of farms recorded indicators CI and DO at an extended level [62% (602 days) and 39% (304 days] respectively, where mature cows (40.03; 23.81) in BCS ≤ 2 (32.91; 19.58) contributed to majority of the recorded CI and DO. Bonsmara breed type (5.56%) in BCS ≤ 2 (6.64%) obtained majority of the overall 12% recorded FC in smallholder farms.

**Table 2:** Description of reproductive performance indicators of participating cows

Variable	Cumulative PR	Cumulative FC	Cumulative DO	Cumulative CI
***Cow age class***				
First	4.82	2.61	5.21	8.11
Second	6.77	0.87	6.40	8.63
Mature	33.62	7.19	23.81	40.03
Aged	5.63	1.63	3.79	5.43
** Overall**	**50.84**	**12.30**	**39.21**	**62.18**
***P*** value	** < .0001**	**0.0003**	**0.3122**	**0.9559**
** Cremer v**	**0.0950**	**0.1438**	**0.0510**	**0.0282**
***BCS***				
** ≤ 2**	21.01	6.64	19.58	32.91
** 3**	27.73	4.68	18.09	25.84
** > 3**	2.10	0.98	1.54	3.43
** Overall**	**50.84**	**12.30**	**39.21**	**62.18**
***P*** value	** < .0001**	**0.0537**	** < .0001**	** < .0001**
** Cremer v**	**0.0950**	**0.0913**	**0.1789**	**0.1515**
***Breed type***				
Nguni type	5.13	0.87	0.45	1.7
Afrikaner type	2.62	0.98	2.38	2.72
Angus type	1.23	0.00	2.16	2.38
Beef master type	8.56	2.40	0.82	0.60
Bonsmara type	20.46	5.56	1.41	3.72
Boran type	0.92	0.00	6.25	1.34
Brahman type	3.84	0.00	1.04	27.46
Drakensberger type	3.57	0.98	1.41	1.17
Hereford type	0.77	0.11	4.17	11.01
Simmental type	3.74	1.40	19.12	10.10
** Overall**	**50.84**	**12.30**	**39.21**	**62.18**
*** P*** value	** < .0001**	**0.1683**	** < .0001**	** < .0001**
** Cremer v**	**0.1531**	**0.1184**	**0.1695**	**0.1450**

Table 3 presents the logistic regression model analysis for association between risk factors and PR. The model predicted increased odds of PR for cows bred between December-March, November-February and January-March [OR = 3.81; 2.561 & 2.695] that are not lactating [OR = 1.28] and in BCS 3 [OR = 1.083] at breeding. Second calvers were predicted to have increased odds [OR = 1.10] of becoming pregnant in herds that practices culling of old cows [OR = 4.18]. The Nguni breed type also projected an increase in the odds [OR = 1.42] of PR. Moreover, bull to cow ratio and the culling of non-productive cows were also identified as factors associated (P ≤ 0.05) with PR.

**Table 3:** The Binary logistic regression model summarizing herd associations between risk factors and the odds of PR in smallholder herds

Variable	SE	OR	95% CI of OR		*P value*
			Lower	Upper	
BCS at breeding					** < *.0001***
BCS 1 vs 4	0.4283	0.260	0.081	0.833	***0.0430***
BCS 2 vs 4	0.1586	0.512	0.346	0.759	***0.2427***
BCS 3 vs 4	0.1560	1.083	0.755	1.555	***0.0003***
*** BCS 4***	***Ref***				
Breed type					** < *.0001***
Beefmaster type vs Bonsmara type	0.1749	0.362	0.192	0.653	0.1768
Simmentaler type vs Bonsmara type	0.1082	0.361	0.225	0.620	0.0421
Boran type vs Bonsmara type	0.2886	0.440	0.213	0.922	0.9266
Brahman type vs Bonsmara type	0.0421	0.432	0.212	0.850	0.0990
Drakensberger type Bonsmara type	0.2431	0.631	0.321	1.271	1.8795
Hereford type vs Bonsmara type	0.3632	0.492	0.190	1.190	0.8546
Nguni type vs Bonsmara type	0.1741	1.420	0.221	0.793	0.6722
*** Bonsmara type***	***Ref***				
**Lactation status**					** < *.0001***
Dry vs Wet	0.05	1.280	1.091	1.501	0.0020
*** Wet***	***Ref***				
**Breeding months**					** < *.0001***
August-October vs September-December	0.4617	0.471	0.132	1.672	0.6097
Continuous vs September-December	0.123	0.390	0.181	0.840	0.0005
December-March vs September-December	0.210	3.812	0.233	0.951	0.32
January-March vs September-December	0.301	2.695	0.060	0.961	0.0109
March-June vs September-December	0.422	1.615	0.212	1.502	0.8356
November-February vs September-December	0.370	2.561	1.464	9.901	< .0001
October–March vs	0.280	0.552	0.251	1.192	0.9362
***September-December***	***Ref***				
**Culling old cows**		4.18	0.472	0.834	< .0001
Culling non-productive cows	0.20	0.47	0.302	0.670	***0.019***
**Cow age class**					***0.05***
First calvers vs Mature cow	0.16	0.716	0.320	0.891	0.1921
Aged cows vs mature cow	0.13	0.959	0.401	0.821	0.2721
Second calvers vs Mature cow	0.07	1.104	0.491	0.880	0.8523
***Mature cow***	***Ref***				
** BCS prior breeding**	0.19	0.471	0.292		0.0802
Bull to cow ratio	0.486	1.242	0.832	1.841	0.0301

Statistically significant at level (*p* < *0.01; p* < *0.05*). *SE* = Standard Error, *OR* = odds ratio, *CI* = confidence interval. *Ref* = Baseline reference variable used as the comparison point for the other categories

Table 4 present risk factors associated with CI in smallholder beef cattle farms. The model predicted an extended CI on cows in BCS 1 [OR = 3.254] and 2 [OR = 3.775] compared to 3 [OR = 1.694]. Cows that experienced pregnancy loss due to abortion had higher odds of having an extended CI [OR = 1.34] as compared to those that gave birth to stillborn calves [OR = 0.47]. Extended CI was higher in cows that had calved in the Autumn [OR = 1.03] compared to the Summer [OR = 0.34] and Spring calving months. Brahman [OR = 2.350], Hereford [OR = 2.073] and Simmentaler breed types [OR = 3.266] had an increase in CI. Moreover, first calvers had increased odds [OR = 4.240] in extended CI. The model further fitted variables BSC prior breeding, keeping calving records and bull to cow ratio as herd management factors associated (P ≤ 0.05) with extended CI.

**Table 4:** The cumulative logit regression model summarizing herd associations between risk factors and the odds of CI in smallholder beef cattle herds

Variable	SE	OR	95% CI of OR		*P value*
			**Lower**	**Upper**	
**BCS prior breeding**					** < *.0001***
**BCS at breeding**					** < .0001**
BCS1vs 4	430.5	3.254	0.186	0.369	0.9765
BSC2vs 4	0.2981	3.775	0.010	0.739	< .0001
BCS 3vs 4	0.2538	1.694	0.137	0.603	0.1439
*** BCS 4***	***Ref***				
**Breed type**					** < *.0001***
Afrikaner Type vs Bonsmara Type	0.3733	0.849	0.469	1.538	0.5889
Angus Type vs Bonsmara Type	0.2982	0.775	0.442	1.359	0.3745
Beefmaster vs Bonsmara Type	0.2784	1.736	1.080	2.792	0.0228
Nguni type vs Bonsmara Type	0.2300	1.482	0.759	2.893	0.0461
Boran Type vs Bonsmara Type	0.4609	1.020	0.471	2.211	0.6478
Brahman type vs Bonsmara Type	0.6679	2.350	1.033	5.343	0.2388
Drakensberger type vs Bonsmara type	0.3124	0.664	0.376	1.173	0.1584
Hereford vs Bonsmara Type	0.5875	2.073	0.681	6.312	0.1995
Simmentaler Type vs Bonsmara Type	0.5377	3.266	0.882	12.100	0.0765
*** Bonsmara type***	***Ref***				
**Cow age class**					***0.0071***
Aged cow vs Matured	0.1977	1.245	0.699	2.220	*0.1385*
First calvers vs Matured	0.2378	4.240	2.105	8.540	< *.0001*
Second calvers vs Matured	0.1195	1.470	0.987	2.189	*0.2873*
Matured	***Ref***				
**Reason for calf loss**					***0.0171***
Aborted	78.2578	1.336	0.055	32.666	0.9834
Stillborn	78.2574	0.478	0.020	11.507	0.9729
**Calving records**	0.4117	3.148	1.405	7.055	0.0514
**Culling Non-productive cows**	0.2761	0.494	0.287	0.848	***0.0106***
**Bull to cow ratio**	0.2784	0.481	0.277	0.833	0.0187
**Calving months**					***0.0006***
Autumn vs Spring	0.2111	1.836	0.179	0.669	0.0034
Winter vs Spring	0.1527	1.744	0.336	1.043	< .0001
Summer vs Spring	0.2935	0.346	0.579	1.838	0.0580
**Spring**	***Ref***				

Statistically significant at level *(p* < *0.01; p* < *0.05*). *SE* = Standard Error, *OR* = odds ratio, *CI* = confidence interval. *Ref* = Baseline reference variable used as the comparison point for the other categories

The risk factors associated with DO are presented in Table 5. Cows in BCS 1 during breeding season [OR = 4.79] and cows that calved in Autumn [OR = 1.092] were likely to result in extended DO. Moreover, the model predicted breed types Simmentaler [OR = 1.077] and Boran [OR = 1.005] to have higher odds of extended DO. Furthermore, aged cows had higher odds of having extended days open [OR = 1.498].

**Table 5:** The cumulative logit regression model summarizing herd-adjusted associations between risk factors and the odds of DO (overlay extended) in smallholder beef cattle herds

Variable	SE	OR	95% CI of OR		*P value*
			**Lower**	**Upper**	
**Calving season**					** < *.0001***
Autumn vs Spring	0.1679	1.092	0.588	2.027	0.8452
Winter vs Spring	0.1021	0.861	0.509	1.456	0.0448
Summer vs Spring	0.1095	0.730	0.428	1.244	0.0007
***Spring***	***Ref***				
**Breed**					***0.0001***
Afrikaner type vs Bonsmara type	0.2661	0.549	0.326	0.924	0.0241
Angus type vs Bonsmara type	0.3313	0.849	0.443	1.625	0.6204
Beefmaster type vs Bonsmara type	0.2109	0.455	0.301	0.688	0.0002
Bonsmara type vs Nguni type	0.1788	0.659	0.465	0.936	0.0198
Boran type vs Bonsmara type	0.3555	1.005	0.501	2.017	0.9889
Brahman type vs Bonsmara type	0.5449	0.318	0.370	3.135	0.8911
Drakensberger type vs Bonsmara type	0.2864	0.199	0.114	0.349	< .0001
Hereford type vs Bonsmara type	0.5336	0.262	0.092	0.745	0.0120
Simmentaler type vs Bonsmara type	0.2499	1.077	0.195	0.520	< .0001
*** Bonsmara type***	***Ref***				
**BCS Prior breeding**	0.2698	0.724	0.427	1.228	0.0188
**Cow age class**					***0.0220***
Aged cows vs Matured cows	0.1515	1.498	0.952	2.357	0.0358
First calvers vs Matured cows	0.1797	0.785	0.465	1.326	0.1268
Second calvers vs Matured cows	0.0962	1.038	0.756	1.425	0.7808
***Matured***	***Ref***				
**BCS at breeding**					***0.030***
BCS breeding 1 vs 4	1.3337	4.792	0.351	65.422	0.2400
BCS breeding 2 vs 4	0.1299	1.094	0.848	1.411	0.4888
BCS breeding 3 vs 4	0.2196	0.523	0.990	2.341	0.0555
***BSC 4***	***Ref***				

Statistically significant at level *(p* < *0.01; p* < *0.05*). *SE* = Standard Error, OR = odds ratio, *CI* = confidence interval. *Ref* = Baseline reference variable used as the comparison point for the other categories

Table 6 presents risk factors associated with FC. The model highlighted an increase in the odds of FC in herds practicing continuous breeding season [OR = 12.86] with cows in BCS of 1 [OR = 4.32] and 2 [OR = 3.059] compared BSC 3 [OR = 0.120] at calving. The odds of FC was high in aged cows [OR = 3.827] and First calvers [OR = 2.218]. Lactation status was further fitted as a factor associated (P ≤ 0.0001) with FC.

**Table 6:** The Binary logistic regression model summarizing herd associations between risk factors and the odds of FC in smallholder beef cattle herds

Variable	SE	OR	95% CI of OR		*P value*
			**Lower**	**Upper**	
**Lactation**					** < *.0001***
**Dry vs Wet**	0.1610	0.710	0.378	1.335	0.2882
**Insemination months**					** < *.0001***
Continuous vs September-December	38.0911	12.86	0.211	85.899	0.9656
December-February vs September-December	38.0914	1.469	0.219	9.874	0.9897
December-March vs September-December	38.0890	1.349	0.075	24.404	0.9897
January-March vs September-December	38.0918	4.250	0.664	250.172	0.9425
March-June vs September-December	38.0970	2.900	0.141	59.548	0.9736
November-February vs September-December	304.7	< 0.001	< 0.001	> 999.999	0.9591
October–March vs September-December	38.0925	3.361	0.372	30.388	0.9706
***September-December***	***Ref***				
**BCS at calving**					***0.0246***
BCS 1 vs 4	0.2921	4.322	1.148	16.272	0.0068
BCS 2 vs 4	0.2921	3.059	0.908	10.308	0.0477
BCS 3 vs 4	0.2508	0.120	0.353	3.557	0.0255
***BSC 4***	***Ref***				
**Cow age class**					
Aged cows vs Matured	0.3103	3.827	1.263	11.591	0.0164
First calvers vs Matured	0.1991	2.218	0.701	7.021	0.5495
Second calvers vs Matured	0.2056	1.286	0.522	3.167	0.0922
***Matured***	***Ref***				

Statistically significant at level *(p* < *0.01; p* < *0.05*). *SE* = Standard Error, *OR* = odds ratio, *CI* = confidence interval*. Ref* = Baseline reference variable used as the comparison point for the other categories

## Discussion

The current study assessed risk factors associated with reproductive performance in smallholder beef cattle herds raised under low input extensive production system. In this system, animal and management risk factors such as BCS, breed type, cow age class, breeding and calving season were identified as major factors associated with the current recorded 50% pregnancy rate, 12% fetal and calf loss and the extended 602 days and 304 calving interval and days open in this research.

The current study has indicated that an increased PR in smallholder farms could be achieved through maintaining breeding cows in BCS of 3 of the1-5 scale during breeding season. These findings were consistent with the study of Kim and Jeong (2019) reflecting that cows with BCS < 3.0 had lower probability of conception than cows with BCS ≥ 3.0. Furthermore, the report by Ayres et al. (2014) indicated that a decrease in BCS < 2.5 is associated with 9% decrease in PR. Additionally, an increase in likelihood of extended DO and CI observed for cows in BCS ≤ 2 in the current study has indicated that maintaining good BCS prior breeding season is as important as maintaining post calving. These results reaffirmed that herd nutrition, particularly during pregnancy and postpartum interval to estrus is a significant determinant of herd reproductive outcomes (Ayres et al. 2014; Nazhat et al. 2021). Adequate nutrition supply in smallholder farms is reported as a major challenge and majority of farmers find supplementation to be an expensive exercise (Meissner et al. 2013). However, one strategy described by da Silva et al. (2017) for low input farmers in Brazil was to supplement pregnant cows in the last trimester of pregnancy to assist in maintaining BCS and body weight post calving and to reduce the cost of supplementation for poor resource farmers. According to their findings, supplemented cows at third trimester of pregnancy tended to exhibit greater weight, approximately 480 kg in comparison to their non-supplemented counterparts (465 kg). Additionally, supplemented cows displayed elevated progesterone concentrations which is a positive indicator of their readiness to enter the estrus phase and cycle successfully (da Silva et al. 2017).

Although it is well known that crop residues have low-quality crude fiber percentages (18%) and low-quality total digestible nutrient percentages (less than 60%), Burrow (2019) had argued that feeding cattle with crop residues during dry periods has been found to be a cost-effective supplementation strategy in smallholder farms. The challenge with smallholder farms might not only be the low quality of feed however, limited amount of feed of any kind (Widiati et al. 2022; Slayi et al. 2023). Residues from cultivated fodder crops including maize, sorghum, millet, barseem, shaftal, soybean, cluster beans and cowpea have been successfully implemented for supplementation during the dry season in the majority of smallholder farms within extensive production systems (Nyaata et al. 2000; Lamidi and Ologbose 2014). These residues are recognized for their higher crude protein levels (Iqbal et al. 2015). Adoption of these residues may be essential for addressing nutritional gaps, improving overall body condition and positively influencing reproductive performance in smallholder farms.

The increased odds of extended CI and DO for Simmentaler and Hereford breed types as compared to Nguni type indicates the significance of farming with adapted breeds in smallholder farms (Mapiye et al. 2019; Jordaan et al. 2021). Moreover, it is noteworthy to mention that the majority of PR was recorded from the Nguni and the Bonsmara breed type in the current study. The distinctive traits of locally adapted breeds that makes them resilient to common challenges faced by smallholder farmers, including diseases, heat stress and limited feed resources have been thoroughly documented (Jordaan et al. 2021; Widyas et al. 2022). Their small to medium frame sizes, exemplified by the Nguni and Bonsmara breed allow them to thrive on minimal nutrient resources found in the grazing veld (Rege & Tawah 1999; Ramsay et al. 2000; Madhusoodan et al. 2019; Gray 2023). In Indonesia, Zuhri et al. (2019) has highlighted that the small frame Madura cattle recorded days open of 134 lower than 168 days recorded in Brahman cross cattle raised in East Java (Zuhri et al. 2019).

Furthermore, in Namibia, Samkange et al. (2019) documented a higher conception rate of 70% in Nguni cattle compared to 64% in the Simmentaler breed within smallholder farms. This suggest that recognizing and promoting the importance of locally adapted breeds in smallholder beef cattle farms may improve reproductive performance as these breeds are inherently suited to the challenges presented by local ecosystems. However, while indigenous breeds excel in harsh conditions, optimal production and reproductive performance require good management. The increased odds of CI among Brahman breed types in the current study highlight this management necessity.

The first calvers in the current study were predicted to have high probability of extended CI and FC. Moreover, Alan and Andrew (2023) in their report has highlighted that cows in parity one had 8 days extended calving interval as compared to cows in parity 2–5. This emphasis that greater attention and better management with cows in this category is significant as they still require cellular maintenance and growth (Temesgen et al. 2022). A mitigating strategy suggested by Orihuela and Galina (2019) is that farmers may implement early weaning at 180 days postpartum as a management tool to manage extended calving intervals in first calf cows, primarily when nutritional demands during lactation are not adequately met. As with first calf cows, farmers need to pay attention to aged cows in the herds. The current study revealed that aged cows resulted in increased odds of extended days open and FC. While the implementation of management strategies such as culling aged or non-productive cows presents challenges in smallholder herds as demonstrated in the current study, it is essential for farmers to adopt this strategy to eliminate poorly performing cows and maintain the productivity and profitability of the herd (Sessim et al. 2020). By analyzing calving data from the past two to three years for individual cows, farmers can identify those ranking in the bottom 10 to 25% for successfully weaning a calf annually. Such candidates should be considered as candidates for culling (Rilanto et al. 2020).

Similar to the current study, Temesgen et al. (2022) emphasized that cows calving in Autumn had more days open (157 days) than cows calving in spring and summer seasons. Moreover, the current study highlighted the highest probability of FC to be associated with continuous breeding season in smallholder herds. These findings validates the importance of a selective breeding season to manage calving season (Pessoa et al. 2018). For improved reproductive outcomes, it is therefore recommended that breeding and calving season should be synchronized with the availability of green fodder, primarily during early lactation (Burrow 2019). Moreover, the report by Kim and Jeong (2019) proposed that for increased survival probability in a cow-calf operation, it is preferable to breed/inseminate cows during the rainy seasons in tropical regions. The latter explains the model predictions of increased PR on breeding seasons December-March, November-February and January-March reported in the current study.

Herd health play a major role in reproductive performance and the findings from the present study were no different (Pérez-Mora et al. 2020). Herds experiencing pregnancy loss due to abortion in the present study exhibited extended calving intervals, highlighting a crucial factor that should be prioritized in management strategic plans for smallholder farms. According to Deresa et al. (2020) infectious agents causing abortions affect follicular growth patterns through reduced growth of dominant follicles in 45 to 85% of breeding cows. Moreover, Wathes et al. (2020) highlighted that infected cows inhibit or delay ovulation mechanisms through decrease luteinizing hormone pulsatility. The delay in oestrus results in prolonged intervals between calving and conception, thus increasing the days open. Bulls are generally known to be the carriers of detrimental pathogens and diseases spread during the breeding season (Njiro et al. 2011; Underwood et al. 2015). Therefore, testing for transmissible disease before breeding season is encouraged in smallholder beef cattle herds.

The findings in the current study revealed evidence supporting the efficacy of adopting an integrated management model for enhancing reproductive permanence in SA smallholder beef cattle farms. A model maintaining cows at an optimal body condition, selecting breeds adapted to local environments, timing breeding activities with favourable seasons, management intervention for first calvers and implementing effective culling strategies may be significant in improving reproductive performance in beef cattle smallholder farms. Farmers could benefit from this research by adjusting breeding practices to capitalize on the highlighted favourable months. Moreover, emphasizing optimal body condition score of three and considering the reproductive impact of culling practices for old and non-productive cows on improvements of herd reproductive outcomes. These strategies may be achieved through consistent recording and monitoring of herd performance for improved management. The current baseline research may be used in policy frameworks in incorporating provisions for monitoring and evaluating the effectiveness of integrated reproductive management interventions. It is therefore, recommended that continuous data collection, analysis and feedback mechanisms will ensure the identification of long-term trends and facilitate ongoing improvement on reproductive performance in smallholder beef cattle farms.

## Conclusion

The assessment of the risk factors on reproductive performance in this study demonstrated that both herd management and animal factors determine the reproductive performance of smallholder farms. The main outcome emphasized that improved management attention on risk factors such BCS, breeding season and breed type may reduce extended CI and DO, and increase PR in smallholder farms as these factors were found to have the most influence on reproductive performance.

## Supplementary Information

Below is the link to the electronic supplementary material.

Supplementary file1 (DOCX 17 KB)

## Data Availability

Not applicable.
