# Supplementary material for: Risk factors underlining reproductive performance in smallholder beef cattle herds of South Africa
Source: Trop Anim Health Prod. 2024 Oct 2;56(8):320. doi: 10.1007/s11250-024-04181-x (PMC11446968; doi:10.1007/s11250-024-04181-x)
Supplement: Supplementary file 1 — Supplementary file1 (DOCX 17 KB) [file 11250_2024_4181_MOESM1_ESM.docx]

**Risk factors underlining reproductive performance in smallholder beef cattle herds of South Africa**

**Tropical Animal Health and Production**

**Marble Nkadimeng ^1,2^*. Este Van Marle-Köster^1^. Nkhanedzeni B. Nengovhela^3,4^.**

**Fhulufhelo V. Ramukhithi^2^. Masindi L. Mphaphathi^2^. Johannes M. Rust^5^.**

**and Mahlako L. Makgahlela^2,6^**

^1^Department of Animal and Wildlife Sciences, University of Pretoria, Hatfield, Pretoria 0002, South Africa

^2^Agricultural Research Council, Germplasm Conservation and Reproductive Biotechnologies,

Private Bag X2, Irene, Tshwane 0062, South Africa

^3^Department of Agriculture, Land Reform and Rural Development, Delpen Building, Corner Annie Botha

and Union Street, Riviera, Pretoria 0001, South Africa

^4^Department of Agriculture and Animal Health, University of South Africa, Florida 1710, South Africa

^5^Döhne Agricultural Development Institute, Stutterheim 4930, South Africa

^6^Department of Animal, Wildlife and Grassland Sciences, University of the Free State,

Bloemfontein 9301, South Africa

***** Correspondence: [nkadimengm1@arc.agric.za](mailto:nkadimengm1@arc.agric.za)

**Supplementary Material**

**Table S1** Phenotype characteristics of beef cattle breeds

| Breed | Phenotypic characteristic | Reference |
| --- | --- | --- |
| Nguni | Multi-colored coats, with many different patterns (white, brown, golden yellow, black, dappled, or spotty)  Black tipped noses are  This small framed breed | Scholtz et al. 2010; Makina et al. 2014 |
| Afrikaner | Medium frame  Yellow to red lateral colour horn with a twist.  typically red which can vary from light tan to deep cherry red | Strydom et al. 2000; Makina et al. 2014 |
| Drakensberger | Medium to large frame breed  Black smooth coat  Strikingly long and deep body with a mild  small shoulder hump | Makina et al. 2014 |
| Bonsmara | Red in colour  Medium to large framed Smooth coated | Muchenje et al. 2009; Bhaveni Kooverjee et al. 2020 |
| Boran | The horns are pointed forward, upright, and short, with a thick base.  Pyramidal shaped hump.  Broad brisket and deep shoulders with wide hindquarters. | Lochner 2018 |
| Simmental | Colour varies from gold to red  White head is white and white band over the shoulders  Pigment around the eyes | https://www.simmentaler.org/Breed-History.htm |
| Hereford | Dark red to red-yellow colour  White head, crest, underline, socks and tail switch | Leesburg *et al.*, 2013 |
| Brahman | Large hump over the top of the shoulder and neck.  Light to medium grey.  Their horns curve upwards | Mukuahima, 2008 |
| Beef Master | Light red to dark red  white mottle on their faces | Mukuahima, 2008 |

^Note: The phenotype characteristics presented in this table is based on available data up to 2020^

**References**

<https://www.simmentaler.org/Breed-History.htm>. Accessed on 28 May 2024.

Kooverjee, B.B., Soma, P., Van Der Nest, M.A., Scholtz, M.M., and Neser, F.W.C., 2022. Selection Signatures in South African Nguni and Bonsmara Cattle Populations Reveal Genes Relating to Environmental Adaptation, Frontiers in Genetics, 13, 1-11. doi: 10.3389/fgene.2022.909012

Leesburg, V.L.R., MacNeil, M.D., van Marle-Köster, E., Mapholi, O., and Neser, F.W.C.. 2013. Impact of Line 1 germplasm on South African Hereford cattle, South African Journal of Animal Science, 43(2), 153- 158. <http://www.scielo.org.za/scielo.php?script=sci_arttext&pid=S0375-> 15892013000200011&lng=en&tlng=en.

Lochner. D. 2018. Phenotypic and genetic characterization of South African Boran cattl, Masters thesis, University of Pretoria, South Africa.

Makina, S.O., Muchadeyi, F.C., van Marle-Köster, E., MacNeil, M.D., Maiwashe, A., 2014. Genetic diversity and population structure among six cattle breeds in South Africa using a whole genome SNP panel. Frontiers in Genetics, 14,1-7. https://doi.org/10.3389/fgene.2014.00333

Muchenje, V., Dzama, K., Chimonyo, M., Strydom, P.E., and Raats, J.G.,2009. Relationship between pre- slaughter stress responsiveness and beef quality in three cattle breeds, Meat Science, 81, 653–657. doi:10.1016/j.meatsci.2008.11.004

Mukuahima, G., 2008. The performance of beef cattle bulls in the Vrede district of Mpumalanga, South Africa, MSc (Agric) Dissertation, University of Pretoria, South Africa.

Scholtz, M.M., and Bester, J., 2010. Off-take and production statistics in the different South African cattle sectors: Results of a structured survey, Applied Animal Husbandry and Rural Development, 3, 19-23.

Strydom, P.E., Naude, R.T., Smith, M.F., Scholtz, M.M., van Wyk, J.B., 2008. Characterisation of indigenous African cattle breeds in relation to meat quality traits, Meat Science 55 (2000) 79-88. PII: S0309-1740(99)00128-X
